# Transcriptome and metabolome analyses reveal phenotype formation differences between russet and non-russet apples

**DOI:** 10.3389/fpls.2022.1057226

**Published:** 2022-11-08

**Authors:** Ziqi Wang, Shasha Liu, Wenping Huo, Min Chen, Yugang Zhang, Shenghui Jiang

**Affiliations:** ^1^ College of Horticulture, Qingdao Agricultural University, Qingdao, China; ^2^ Engineering Laboratory of Genetic Improvement of Horticultural Crops of Shandong Province, Qingdao, China; ^3^ Yantai Institute of Coastal Zone Research, Chinese Academy of Sciences, Yantai, China

**Keywords:** transcriptome, metabolome, apple, fruit russeting, suberin

## Abstract

The apple is an economically important fruit, and fruit russeting is not conducive to its appearance. Although studies have examined fruit russeting, its mechanism remains unclear. Two apple strains of the F_1_ hybrid population derived from ‘Fuji’ and ‘Golden Delicious’ were used in this study. We found that the skin of russet apples was rough and fissured, while that of non-russet apples was smooth and waxy. Chemical staining, LC- and GC-MS showed that both lignin and suberin were increased in russet apple skin. Meanwhile, genes involved in lignin and suberin synthetic pathways were upregulated in russet apple skin. Additionally, we found many differentially expressed genes (DEGs^1^) involved in hormone biosynthesis and signaling and stress responses in the two apple strains. We found that WRKY13 may influence russeting by regulating lignin synthesis. Our study identified several candidate metabolites and genes, which will provide a good foundation for further research.

## Introduction

Apples are one of the most commonly consumed fruits in everyday life and are preferred by the public for their sweet and sour tastes. According to FAO 2020 (https://www.fao.org/faostat/en/#data/QCL/visualize), global apple exports reached 8.2 million tons in 2020, which holds great economic importance. In addition to taste and aroma, fruit appearance is an important criterion in apple evaluation. The skin of a normal apple is smooth and waxy, and the primary skin consists of cuticle, epidermis, and hypodermis (Khanal et al., 2013). Compared with normal fruits, russet fruits show yellow-brown rust spots on their surface.

Fruit russeting is a secondary metabolic process, where cuticle cracks and suberin rapidly form to replace the broken cuticle to prevent water dissipation and reduce pathogen infection (Lashbrooke et al., 2015). The causes of fruit russeting have been studied in fruit crops, such as apple, pear, and grape (Lashbrooke et al., 2015; Huang et al., 2020; Zhang et al., 2021). Apple russeting is influenced by environmental factors, and a prolonged period of moisture on the surface of the fruit skin or high humidity in the environment can cause the skin to crack, ultimately leading to russeting (Knoche et al., 2011).

With the development of omics technologies, fruit russeting research has shifted from physiological characteristics to molecular mechanisms. Transcriptome, metabolome, and proteome association analyses identified differences in suberin, phenylpropane, cutin, and wax biosynthesis between russet and non-russet pears (Shi et al., 2021). Therefore, it is speculated that phenylpropane and cuticle metabolism have important effects on fruit russeting. LC-MS and nuclear magnetic resonance identified three triterpene-caffeates extracted from russet apple skins of the ‘Merton Russet’ cultivar as oleanolic acid-3-trans-caffeate, betulinic acid-3-cis-caffeate, and betulinic acid-3-trans-caffeate, which were only found in russeting fruit (Andre et al., 2013). Triterpenes are included in the epicuticular and intracuticular wax of plant cuticles (embedded in cutin polymers). In apples, triterpenes are present in the cuticular skin layer, where they account for 60% of the wax content (Zhang et al., 2020). Thus, the presence of triterpene caffeates disrupts cuticle formation, which initiates fruit russeting. Furthermore, the transcription factor MYB66 can bind to *OSC5* to regulate triterpene caffeate formation, which disrupts normal cuticle synthesis and leads to fruit russeting in apple (Falginella et al., 2021). In apples, skin suberization has been linked to the presence of triterpene compounds (Legay et al., 2016). In addition, transcriptome results showed that suberin synthesis-related genes are abundantly expressed in russet fruits, including *LACS*, *FAR*, *KCS*, *CYP86*, *GPAT*, *ASFT*, *ABCG*, and *LTP* (Zhang et al., 2021). Transcription factors are also involved in suberin accumulation in fruit russeting, which is regulated by MYB93 in apple peels, thereby affecting fruit russeting (Legay et al., 2016). In pears, lignin also demonstrated an important effect on russeting because of its high expression of *PAL*, *4CL*, *HCT*, *C4H*, *COMT*, and *CcoAOMT* (Wang et al., 2020). In addition, *CCR* and *CAD4-* and *CAD9-like* genes were downregulated in russet apples (Legay et al., 2015). In addition to the upregulation of lignin synthesis genes, a substantial accumulation of phenolics related to lignin synthesis has been identified in russet grape fruits (Huang et al., 2020). Moreover, the genetic factors involved in fruit russeting were analyzed. The main effector gene controlling russet variation in ‘Renetta Grigia di Torriana’ apples is located on chromosome LG12 (Falginella et al., 2015). The *PpRus* gene, which controls the pear russet skin trait, was identified *via* Bulked Segregant analysis and is located on chromosome 8 of the pear genome (Ma et al., 2021).

In this study, we found that the skin of russet apples had more lignin and suberin than that of non-russet apples. Metabolome analysis showed that metabolites related to lignin and suberin synthesis in russet apple skin were higher than those in non-russet apple skin. Transcriptome data also demonstrated that the genes involved in the lignin and suberin synthetic pathways were upregulated in russet apple skin. Interestingly, we also found that a WRKY transcription factor, WRKY13, may influence russeting by regulating lignin synthesis. Our findings provide several candidate metabolites and genes to understand fruit russeting.

## Materials and methods

### Plant materials

Russet and non-russet apples were obtained from two strains of the F_1_ hybrid population derived from ‘Fuji’ and ‘Golden Delicious’ in 2020. Apple trees were cultivated in the Jiaozhou Agricultural Science and Technology Demonstration Garden of Qingdao Agricultural University (Jiaozhou, Shandong Province). We chose fruits 120 days after full bloom (DAFB) as the material for our experiments because of their different phenotypes. Both fruit types were grown under the same conditions. Each type of fruit was similar in shape and size, without any mechanical damage or disease. The skin tissue was collected, immediately frozen in liquid nitrogen, and stored at -80 °C for further use. Fresh fruit pericarps (0.5 × 0.5 cm) were fixed in FAA fixative for microscopic observation.

### Microscopy

A portion of the fruit peel was cut into paraffin sections, and the samples were sliced into 10 µm-thick sections using a Leica RM2245 paraffin slicer. Phloroglucinol HCl (Wiesner) was used for lignin deposition. Sudan red 7B was used to stain the deposited suberin. The stained sections were observed under a Leica microscope. Samples observed *via* scanning electron microscopy (SEM) were cut into small pieces (0.5 × 0.5 cm), fixed using FAA, and dehydrated through an acetone gradient. The samples were then subjected to critical-point drying and gold spraying. The fabricated samples were then subjected to SEM analysis.

### GC-MS sample extraction and determination

The peels were ground into a powder. Fruit peel (50 mg) was extracted with 150 μL of methanol, 200 μL of methyl tert-butyl ether, and 50 μL of 36% phosphoric acid. The solution was vortexed for 3 min and then centrifuged at 12000 r/min for 5 min at 4°C. Then, 200 μL of supernatant was dried on a nitrogen blower, and 300 μL 15% boron trifluoride methanol was added. The solution was vortexed for 3 min and incubated at 60°C for 30 min. Five hundred microliters of n-hexane and 200 μL of saturated NaCl were added to the solution at room temperature. Then, the solution was vortexed for 3 min and centrifuged at 12000 r/min for 5 min at 4°C. The n-hexane layer (100 μL) was used for further analysis. The sample derivates were analyzed using a GC-EI-MS system (GC, Agilent 8890; MS, 5977 B System). The analytical conditions were as follows, GC: column, DB-5MS capillary column (30 m to 0.25 mm × 0.25 μm, Agilent); carrier gas: high purity helium (purity > 99.999%); the heating procedure commenced at 40 °C (2 min), 30 °C/min to 200 °C (1 min), 10 °C/min to 240 °C (1 min), 5 °C/min to 285 °C (3 min); traffic: 1.0 mL/min; inlet temperature, 230 °C; injection volume: 1.0 μL. The EI-MS procedure utilized a Agilent 8890-5977B GC-MS System, temperature: 230°C, ionization voltage: 70 eV; transmission line temperature: 240 °C, four-stage rod temperature: 150 °C, solvent delay: 4 min, scanning mode: SIM. Fatty acids and their metabolites were detected using MetWare (http://www.metware.cn/) on an Agilent 8890-5977B GC-MS platform.

### LC-MS sample extraction and detection

Fruit peels (100 mg) were ground into powder in liquid nitrogen, and 500 μL of 80% methanol was added. The solution was vortexed, incubated for 5 min on ice, and then centrifuged at 12000 r/min for 20 min at 4°C. The supernatant was diluted with mass spectrometry-grade water and then centrifuged at 12000 r/min for 5 min at 4°C. The second supernatant was used for LC-MS analysis. Separation was performed using an Xselect HSS T3 column (2.5 μm, 2.1 × 150 mm). The mobile phase was 0.1% aqueous formic acid solution (solvent A) and 0.1% formic acid in-acetonitrile (solvent B) at a flow rate of 0.4 ml min^–1^. The linear gradient of phase B was as follows: 0–2 min, 2%; 2–15 min, 2%; 15–17 min, 100%; 17-7.1 min, 2%; 17.1–20 min, 2%. Mass spectrometry conditions for positive ions were as follows: curtain gas: 35 psi; collision gas: medium; IonSpray voltage: 5500 V; temperature: 550°C; Ion Source gas: 1:60; Ion Source gas: 2:60. 2. Mass spectrometry conditions for negative ions were as follows: curtain gas: 35 psi; collision gas: medium; IonSpray voltage: -4500 V; temperature: 550°C; ion source gas 1: 60; ion source gas 2: 60.

Based on the Novogene database, multiple reaction monitoring was used to detect the samples. The compounds were quantified using their daughter ions and characterized by retention time, declustering potential, and collision energy. SCIEX OSV1.4 software was used for integration, correction, and analysis.

### RNA extraction, cDNA library construction, and qRT-PCR determination

Total RNA was extracted from apple fruit peels using the RNAprep Pure Plant Kit (Tiangen, Beijing, China), according to the manufacturer’s instructions. RNA concentration and quality were assessed using an Agilent 2100 instrument (Agilent Technologies, Santa Clara, CA, USA). Then, cDNA libraries were constructed for Illumina sequencing, following the manufacturer’s protocol. The libraries were sequenced on an Illumina PE150 platform (Illumina, San Diego, CA, USA). After removing the adapters and low-quality reads, the clean reads were aligned to the reference genome (https://iris.angers.inra.fr/gddh13/index.html) using the Hisat2 software. Gene expression was analyzed using the Counts and StrintTie tools. Differentially expressed genes (DEGs) were identified using the DESeq2 package. RNA-seq included three biological replicates for each sample. Transcriptome data were uploaded to the NCBI Short Read Archive (https://www.ncbi.nlm.nih.gov/sra/) under the accession number PRJNA871277.

First-strand cDNA was synthesized from RNA samples using the PrimeScript™ RT Reagent Kit (Takara RR047A, Japan), and cDNA was used as a template for gene expression analysis. Gene expression was measured using qRT-PCR on a CFX96 Touch Real-Time PCR Detection System (Bio-rad, CFX96 touch). The qRT-PCR was carried out with ChamQ SYBR Color qPCR Master Mix (Vazyme, China), primers, cDNA, and RNase-free water in a total volume of 20 μL. The primers used for qRT-PCR are listed in **Table S1**. Relative express was calculated by the cycle threshold (Ct) 2^−ΔΔCt^ method (Livak and Schmittgen, 2001). The independent biological experiments were performed in triplicates for each sample.

### Statistical analysis

Each result is the mean of three biological replicates for each experiment. One-way analysis of variance was performed *via* Student’s t-test using the GraphPad Prism software. *P* values < 0.05 were considered significant.

## Results

### Microstructural peel observation and comparison of lignin and suberin content between russet and non-russet apples

**Figure 1:**
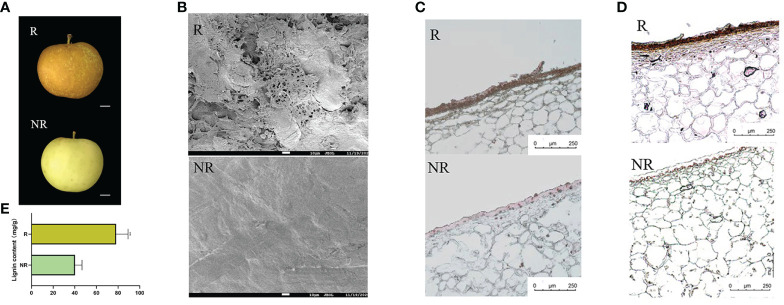
The different phenotypes between russet and non-russet apples at 120 DAFB. **(A)** Fruit phenotypes of the two apples. **(B)** SEM images of russet and non-russet apple fruit skins (scale bars: 10 μm). **(C)** Sudan red 7B staining of russet and non-russet fruit skins (scale bars: 250 μm). **(D)** Phloroglucinol staining (reddish color) of fruit skins (scale bars: 250 μm). **(E)** Lignin content between russet and non-russet fruits. Significance was calculated according to Student’s *t-test*: **P < 0.01.

Russet and non-russet apples were harvested 120 DAFB. Russet fruits had yellowish-brown rust spots on their pericarp surface, whereas non-russet fruits had a smooth surface with an intact cuticle (**Figure 1A**). To further investigate the structural differences in the pericarp, we observed the microstructure of the pericarp of both fruits using SEM. The russet fruit pericarp appeared fissured and had a laminar build-up, whereas the non-russet fruit pericarp had a smooth surface with a complete waxy texture (**Figure 1B**). The fruit peel was chemically stained to further analyze russeting composition. The sections were stained to determine the content and location of the peel, and Sudan Red 7B staining was used to determine the suberin content. High suberin content was observed in the russet sample (**Figure 1C**), and the lignin staining was darker in russet peels than in non-russet peels (**Figure 1D**). Moreover, lignin content was significantly higher in russet peels than in non-russet peels (**Figure 1E**).

### Differential gene expression analysis and validation of RNA-seq data

**Figure 2:**
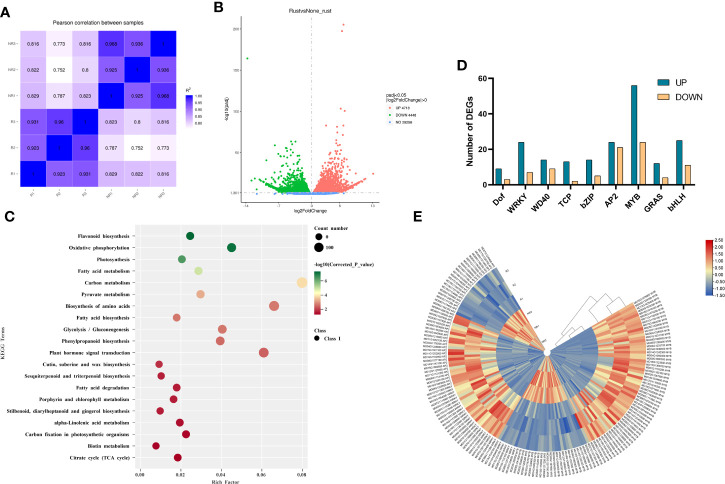
Identification and functional characterization of the DEGs in the two fruits. **(A)** Heatmap showing Pearson correlation coefficients between two samples. **(B)** Volcano plot filtering identified DEGs in the russet and non-russet fruit skin. **(C)** KEGG enrichment analysis of the DEGs in the two fruits. **(D)** Classification of transcription factors. **(E)** Expression heatmap of DEGs related to MYBs, WRKYs and AP2s.

As the fruits of the two strains exhibited different peel phenotypes, RNA-seq was performed to investigate the differences in gene expression between russet and non-russet peels. RNA-seq detected a total of 46,558 known genes and 704 new genes. The Pearson coefficient between biological replicates of all samples was greater than 0.75 (**Figure 2A**), which can be considered a high correlation coefficient between samples with correlated expression patterns, and the experimental results could be used to analyze DEGs. Screening based on |log_2_(FoldChange)| >1 and false discovery rate (FDR) ≤ 0.05, we identified 4718 genes with upregulated expression and 4448 genes with downregulated expression compared to non-russet fruit (**Figure 2B**).

Gene Ontology (GO) analysis showed DEG enrichment in molecular function, biological processes, and cellular components (**Figure S1**). The 20 terms with the highest number of enriched genes in the three ontologies were selected for analysis. For biological processes, genes were enriched in single-organism signaling (143), signal transduction (143), signaling (143), single-organism biosynthetic process (130), lipid metabolic process (120), and other terms. For cellular components, genes were enriched in the protein complex (105), ribonucleoprotein complex (94), and intracellular ribonucleoprotein complex (93). For molecular function, genes were enriched in ADP binding (184), cofactor binding (140), heme binding (133), and tetrapyrrole binding (133).

The DEGs were mapped to the Kyoto Encyclopedia of Genes and Genomes (KEGG) pathway database (**Figure 2C**). KEGG enrichment analysis showed that the DEGs were significantly enriched in stilbenoid, diarylheptanoid, and gingerol biosynthesis (ko:mdm00945), flavonoid biosynthesis (ko:mdm00941), phenylpropanoid biosynthesis (ko:mdm00940), alpha-linolenic acid metabolism (ko:mdm00592), cutin, suberine, and wax biosynthesis (ko:mdm00073), fatty acid degradation (ko:mdm00071), fatty acid biosynthesis (ko:mdm00061), and fatty acid metabolism (ko:mdm01212).

Transcription factors play important roles in plant growth and development by activating or inhibiting gene expression. DEG analysis identified 279 transcription factors, including nine common transcription factor families: Dof, WRKY, WD40, TCP, bZIP, AP2, MYB, GRAS, and bHLH (**Figures 2D, E**). Among them, MYB, WRKY, and AP2 were abundantly expressed in russet fruits, and we speculate that they may play an important role in fruit russeting. In the MYB transcription factor family, 56 genes were upregulated, and 24 genes were downregulated in russet fruit. *MYB6*, *MYB36*, *MYB66*, *MYB67*, *MYB73*, *MYB84*, *MYB93*, and *MYB4*, genes involved in suberin biosynthesis, were upregulated. In the WRKY transcription factor family, 24 transcription factors were upregulated, and seven were downregulated in russet apple. Furthermore, *WRKY13*, *WRKY24*, *WRKY51*, *WRKY56*, and *WRKY74* were significantly upregulated. In addition, 24 transcription factors were upregulated, and 21 were downregulated in the AP2 transcription factor family, these differential expressions in russet apples play a role in fruit russeting formation.

### LC-MS and GC-MS profiling

**Figure 3:**
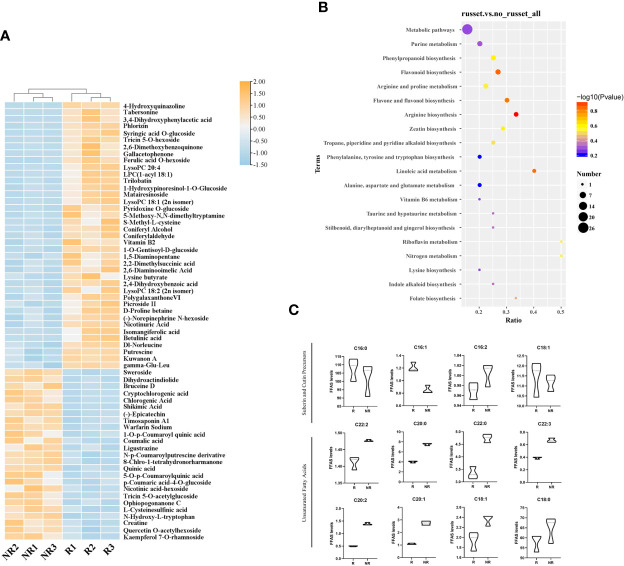
DAM analysis in russet and non-russet apples. **(A)** The heatmap shows the DAMs identified using LC-MS. Orange represents upregulated DAMs, and blue represents downregulated DAMs. **(B)** KEGG enrichment analysis of the DAMs in the two fruits. **(C)** Metabolite contents in the two fruits.

To better explore the differentially accumulated metabolites (DAMs) between russet and non-russet pericarps, fruit peels were subjected to metabolome profiling *via* LC-MS and GC-MS. The DAMs were analyzed using the following parameters: VIP > 1, fold change > 1.5 or < 0.667, and *P* value < 0.05. In total, 165 DAMs were identified, of which 96 and 69 were upregulated and downregulated in russet apple, respectively (**Figure 3A**). All DAMs were divided into 20 categories (**Table S2**). GC-MS was used for targeted determination of the fatty acid content. A total of 32 fatty acids were detected, including medium- and long-chain fatty acids, 16 of which are involved in peel synthesis.

KEGG pathway enrichment analysis showed that the top enriched terms were metabolic pathways (map01100), purine metabolism (map00230), phenylpropanoid biosynthesis (map00940), flavonoid biosynthesis (map00941), and tropane, piperidine, and pyridine alkaloid biosynthesis (map00960) (**Figure 3B**). The results indicated that DAMs from different categories played important roles in fruit russet formation.

Interestingly, we found significant accumulation of cinnamic acid and its derivatives, phenylpropanoids, and phenolic acids in russet apples (**Table S2**). However, flavonoids, amino acids, and their derivatives were significantly enriched in both samples. Therefore, we noticed that during russeting formation, the plant secondary metabolism strengthened, and the metabolic changes in some amino acids and the flavonoids synthesis process also changed.

According to the GC-MS results (**Figure 3C**), we detected the main metabolites involved in cutin, suberine, and wax biosynthesis and biosynthesis of unsaturated fatty acids pathways in pericarp. C16:0 (palmitic acid), C16:1 (cis-9-palmitoleic acid), C16:2 (hexadecanedioic acid), C18:1 (cis-9-octadecenoic acid), and C22:2 (cis-13,16-docosadienoic acid) take part in suberin biosynthesis. C16:0, C16:1, and C18:1 were highly abundant in russet fruits, whereas C22:2 and C16:2 accumulated more in non-russet fruits. C20:0 (arachidic acid), C22:0 (behenic acid), C22:3 (erucic acid), C20:2 (cis-11,14-eicosadienoic acid), C20:1 (cis-11-eicosenoic acid), C18:0 (stearic acid), and C18:1 (trans-9-octadecenoic acid) participate in very long-chain fatty acid biosynthesis, thereby promoting wax synthesis and accumulation. The high levels of these substances may contribute to wax formation in non-russet fruits. The higher accumulation of waxy synthetic precursors in non-russet fruit, compared with russet fruit, suggests that the non-russet pericarp can form cuticles normally. We speculate that fruit russeting is a complex process that combines suberin accumulation and cuticle breakage.

### Identification of weighted gene co-expression network analysis modules associated with russet and non-russet fruits

**Figure 4:**
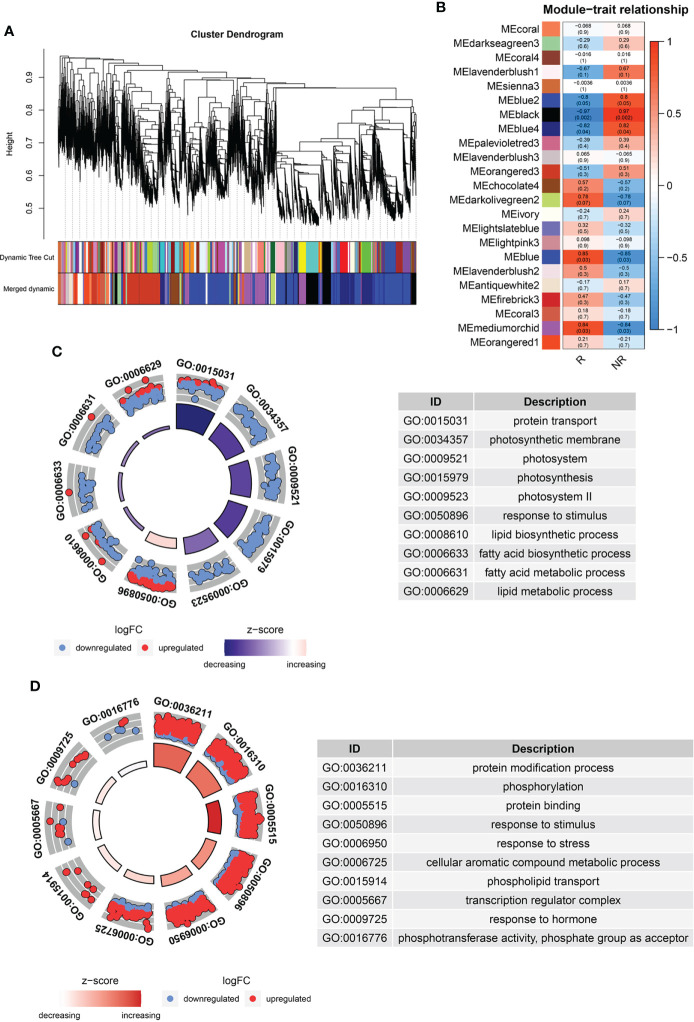
WGCNA analysis of DAMs and DEGs. **(A)** Dendrogram with co-expressed gene modules. **(B)** Module-trait correlations and *P* values. The left panel shows 23 modules in different colors, and the right panel from blue to red represents the correlation between modules. GO analysis of blue2, blue4 and black modules **(C)**, blue and mediumorchid modules **(D)**.

To identify the different gene expression and metabolite modules in the two kinds of fruits, we carried out a WGCNA of all DEGs with metabolites. The results identified 23 co-expression modules labeled with different colors in the cluster dendrogram (**Figure 4A**). Five modules (blue, blue2, blue4, black, and medium orchid) showed a significant correlation between the genes and metabolites (*P* ≥ 0.05; **Figure 4B**). GO analysis of genes in the blue2, blue4 and black modules showed that lipid biosynthesis, lipid metabolism, fatty acid biosynthesis, and fatty acid metabolism were negatively related to fruit russeting (**Figures 4C**, **S2B, D, E**). This may be due to the breakdown of the peel cuticle in russet apples and the decrease in apple wax. We also found that some components of the photosystem (**Figures 4C**, **S2B, D, E**), indicating that light signals were involved in fruit russeting. Interestingly, the enriched processes detected in the blue and mediumorchid modules included phosphorylation and protein modification process, suggesting that protein modification may contribute to fruit russeting (**Figures 4D**, **S2A, C**). Furthermore, blue and mediumorchid modules also included response to stimulus, response to stress and response to hormone (**Figures 4D**, **S2A, C**), indicating that the environment and plant hormone may affect the formation of fruit russeting.

### Combined transcriptome and metabolome analysis and RNA-seq data validation

**Figure 5:**
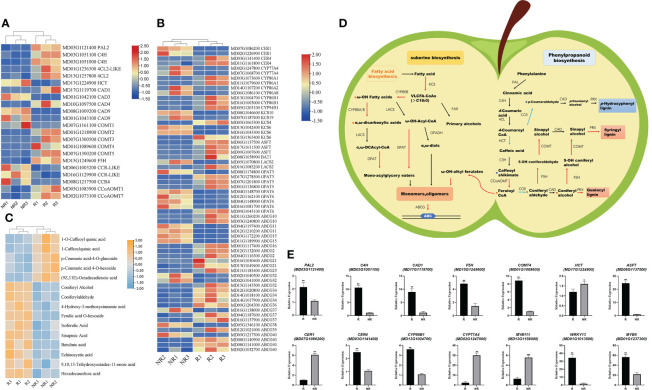
Integrated analysis of metabolomic and transcriptomic profiling. **(A)** DEGs involved in the lignin synthesis pathway. **(B)** DEGs involved in the suberin and cutin synthesis pathway. **(C)** Potential DAMs involved in fruit russeting. **(D)** Proposed model of russeting in apples (Red and blue arrows indicate upregulated and downregulated genes in the pathway, respectively; red and blue circles indicate the increased and reduced metabolites, respectively). **(E)** qRT-PCR validation of the transcriptome data; significance was calculated according to Student’s *t-*test: *P < 0.05, **P < 0.01.

Apple fruit russeting involves many biosynthetic processes, including the suberin and lignin biosynthesis pathways (Jiang et al., 2022). We focused on the cuticle and suberin biosynthesis pathways, as well as phenylpropanoid metabolism. Moreover, the russeting process is regulated by several enzymes and genes. Based on the RNA-seq results, we identified 22 DEGs related to lignin biosynthesis and 62 DEGs related to suberin biosynthesis (**Figures 5A, B**).

The results showed that *PAL, C4H, 4CL, HCT, CCR, CAD, COMT*, and *CCoAOMT* were significantly differentially expressed (**Figure 5A**). Through LC-MS analysis, we found that phenylalanine, caffeic acid, caffeyl aldehyde, coniferyl aldehyde, and coniferyl alcohol were significantly enriched in russet samples (**Figure 5C**). Interestingly, 1-Caffeoylquinic acid and the gene expression of *hydroxylcinnamoyl-CoA shikimate/quinate hydroxycinnamoyl transferase (HCT)* were both down-regulated in russeting fruits. The *HCT* is associated with *p-coumarate 3-hydroxylase (C3H)* and diverts the pathway from H-lignin to G- and S-lignin (Wagner et al., 2007). Based on the significant expressions of coniferyl aldehyde, coniferyl alcohol and sinapinic acid, the lignin type in fruit russeting is more likely to be G-S lignin.

We also detected some genes synthesized with suberin biosynthesis. The expression of *CER4*, *CYP86A1*, *CYP86A2*, *CYP86B1*, *CYP86B1*, *CYP86B2*, *ASFT*, *KCS4*, *GPAT5*, *GPAT6*, and *ABCGs* were up-regulated. However, *CER1*, *KCS6*, *KCS10*, *KCS19*, *CYP94B1*, *CYP86A2*, *CYP86A8*, *CYP77A4* and *GPAT3* were down-regulated (**Figure 5B**). We are also concerned about the differential accumulation of metabolites in suberin and cuticle, we found in LC-MS results high accumulation of betulinic acid, echinocystic acid, (9,10,13)-trihydroxyoctadec-11-enoic acid, and hexadecanedioic acid in russet fruit, while (9Z,11E)-octadecadienoic acid decreased. In addition, hexadecanedioic acid accumulated significantly. We identified significant *ASFT* expression with a high content of feruloyl-CoA (**Figure 5B**). ASFT usually catalyzes feruloyl CoA conversion into ω-OH-alkyl ferulates. The results showed a positive correlation between lignin synthesis and suberin content in this sample. The upregulated *C4H* expression is crucial. C4H greatly affects lignin synthesis and plays an important regulatory role in early lignin G/S differentiation (Hou et al., 2022). The downregulated *HCT* expression and 1-caffeoylquinic acid accumulation were also well demonstrated. In addition, the upregulated *COMT* and *CCoAOMT* expression also increased ferulic acid and coniferaldehyde accumulation and promoted lignin synthesis in russet fruit. In the suberin and cuticle synthesis pathway, we identified upregulated genes that promote suberin synthesis, including *CER4*, *CYP86As*, *ASFT*, and *KCS4*. Interestingly, some genes were downregulated in this pathway (mdm00730), such as *CER1* and *KCS6*, accompanied by downregulated expression of long-chain fatty acid synthesis precursors (**Figure 5D**). We speculate that lignin and suberin synthesis increased with fruit russeting, whereas cuticle and wax synthesis decreased.

We then performed qRT-PCR to determine the expression of these 12 genes and found that the quantitative results were consistent with the transcriptome results, indicating that the transcriptome results were credible (**Figure 5E**).

### Plant hormone signals and expression of stress-related genes

**Figure 6:**
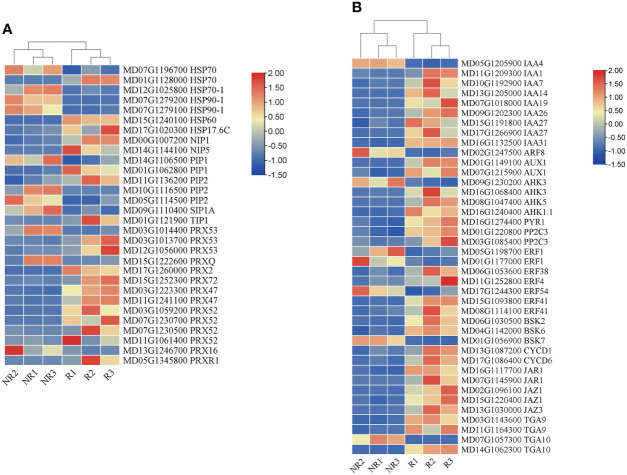
Expression heatmap of DEGs related to stress responses **(A)** and phytohormones **(B)** between the two fruit skins.

Fruit russeting is affected by the environment and hormones. In this study, genes involved in hormone signaling and stress responses were analyzed in addition to the genes related to lignin and suberin biosynthesis. We identified the expression of multiple phytohormone pathway genes (**Figure 6A**). The results showed that the auxin synthesis pathway genes *IAA* and *AUX* were highly expressed in russet fruits. The cytokinin (CK) pathway genes *AHK* and *PYR1* were also expressed at higher levels in russet samples. *PP2C* in the abscisic acid (ABA) pathway was highly expressed in russet samples. *BSK* and *CYCD* were highly expressed in the brassinolide (BR) pathway. In addition, *JAR1, JAZ1, TGA9*, and *TGA10* were upregulated and involved in the methyl jasmonate (MeJA) pathway. Interestingly, some ERFs (*ERF1* and *ERF54*) in the ethylene synthesis pathway were downregulated in the russet samples.

To study the response of russet fruits to external environmental stimuli, we analyzed gene expression abundance under abiotic stress (**Figure 6B**). Heat shock proteins (HSPs), aquaporins (AQPs), and peroxidases (PRXs) exhibited different expression levels. We found that HSP (HSP70*, HSP70-1*, and *HSP90-1*), AQP (*PIP1, PIP2, SIP1*, and *TIP1*), and PRX (*PRXQ, PRX16, PRX47, PRX52, PRX53*, and *PRX72*) genes were upregulated in russet fruits.

## Discussion

### Physiological characteristics and composition analysis of fruit russeting

The skin of russet fruits is brown, rough, and covered with cracks. SEM showed that the cuticle was dramatically reduced, leading to a russet phenotype. For example, fruit russeting gradually appeared as cracks during the development period, although the integrity of the russeting cuticle was still maintained at 31 DAFB in apples (Falginella et al., 2021). SEM observations revealed V-shaped cracks in the peel of 150 DAFB russet apple (Yuan et al., 2019). Non-russet pears were smooth, with no cracks, but cuticle cracks were evident in russet pears (Shi et al., 2021). In this study, we found that russet apple skin was rough, whereas that of other apple was smooth (**Figure 1A**). SEM showed that the russet fruit surface was covered with cracks and gaps and the cuticle was broken, while the waxy layer of the non-russet peel was intact (**Figure 1B**). In addition, sections of russet kiwifruit and pear pericarp showed higher suberin and lignin contents than those of non-russet fruit (Wang et al., 2020; Macnee et al., 2021). These findings are consistent with the results of our study.

In previous studies, metabolome profiling of sand pears revealed that phenols, lignin, ω-unsaturated fatty acids, α,ω-diacids, ω-OH fatty acids, and glycerides were enriched in russet pears (Shi et al., 2021). These are important components of suberin and lignin. In ‘Sunshine Muscat’ grapes, lignin and quercetin play a role in berry russeting (Huang et al., 2020). Ursane-type triterpenes are predominant in waxy apple cuticles, but they shift towards lupane-type triterpenes with russet formation (Falginella et al., 2021). In this study, we detected many compounds that may influence fruit russeting. We found that C16:0, C16:1, and C18:1 contents were high in russet fruits, while C22:2 and C16:2 were higher in non-russet fruits (**Figure 3C**). This indicated that russeting decreased long-chain fatty acid biosynthesis and increased suberin biosynthesis. Moreover, two lignin synthesis-related metabolites, ferulic acid and coniferaldehyde, accumulated in russet apples in this study (**Figure 5C**). These results demonstrate that higher lignin and suberin contents contribute to fruit russeting in apples.

### Molecular mechanism regulating fruit russeting

RNA-seq analysis was performed to reveal the molecular regulatory basis underlying russet fruits with contrasting phenotypes. Previous studies have shown that key genes, such as *CYP, KCS, GPAT, FAR*, and *ABCG*, play an important role in peel russeting (Pollard et al., 2008). In addition, key genes of the phenylpropane metabolism pathway also have an important effect on fruit russeting (Hou et al., 2022). The AP2-type transcription factor MdSHN3 promotes cuticle formation and inhibits fruit russet formation in apple pericarps (Lashbrooke et al., 2015). The transcription factor MdMYB66 can bind *MdOSC5* to promote lupane-type triterpene formation in russet fruits while reducing the ursane-type and oleanane-type formation, causing failure of the early development of apple cuticles and increasing fruit russeting (Falginella et al., 2021). A study on russet apple peels found that the normal development of apple fruit skin produces lenticels, which produce suberin (Straube et al., 2021). Moreover, the chemical phenotypes observed in most of the studied suberin mutants suggest the involvement of partially redundant enzymes from the CYP, GPAT, FAR, and KCS families (Li et al., 2007). In our study, many genes showed strong connections with suberin biosynthesis. For example, we found that *KCS1*, *KCS2*, and *KCS4* were upregulated, while *KCS6, KCS10, KCS11*, and *KCS19* were downregulated (**Figure 5B**). *KCS2* and *KCS20* promote suberin deposition in the periderm, whereas *KCS6* and *KCS11* promote wax formation (Lee et al., 2009; Lian et al., 2021; Li et al., 2021; Huang et al., 2022). These results indicate that the different expressions of *KCS2* and *KCS6* in our study may cause suberin increase and wax reduction. In addition, *PbABCGs* were always expressed at a high level in the ‘Dangshan Su’ russet pear mutant during different development periods (Hou et al., 2018). A putative ABCG family transporter has been identified near a significant quantitative trait locus (QTL) for apple fruit russeting on LG12, which is not collinear with LG8 in sand pear (Falginella et al., 2015). *ABCG2*, *ABCG6*, and *ABCG20* are involved in suberin synthesis in the roots and seed coats (Yadav et al., 2014). In this study, we identified 13 ABCGs with different expression patterns. The upregulated expression of *ABCG2*, *ABCG21*, *ABCG23*, *ABCG34*, *ABCG36*, *ABCG37*, *ABCG39*, and *ABCG40* in russet apples may promote fruit russeting (**Figure 5B**). GPAT is a major determinant of cuticle and suberin composition, catalyzing glycerol-3-phosphate acylation (Yang et al., 2012). *GPAT4* and *GPAT6* primarily esterify acyl groups at the sn-2 position of glycerol-3-phosphate during *Arabidopsis* cutin biosynthesis (Yang et al., 2010). In our study, GPAT6 expression was lower, suggesting that cutin synthesis is attenuated in russet fruit (**Figure 5B**).

Lignin is an important secondary metabolite that affects fruit russetting. PAL is a key enzyme in lignin synthesis, and the transcription factor LIM can interact with PAL-like enzymes to promote lignin synthesis in fruit russeting (Yuan et al., 2019). Studies on ‘Dangshan Su’ pear mutant varieties showed that increased *CCoAOMT* expression resulted in increased lignin content in the outer skin, which led to russet formation (Heng et al., 2016). Research on russet apples has highlighted that lignin synthesis genes, such as *COMT* and *C4H*, are expressed more frequently, contributing to russeting (Legay et al., 2015). In our study, we also found that the lignin synthesis genes *PAL*, *C4H*, *F5H*, *COMT*, and *CCoAOMT* were upregulated in russet apples. This indicated that lignification is an important process in fruit russeting. In addition, *CCR* and *CAD9* expression in russet apples was downregulated, consistent with previous results (Legay et al., 2015).

### Fruit russeting in response to external environmental stress

Environmental factors, such as light, temperature, moisture, and endogenous hormones, influence russeting. In a previous study, light intensity influenced fruit russeting by affecting the levels of endogenous gibberellins (Noè and Eccher, 1996). In pears, rainfall treatment aggravated russeting both in ‘Zaoshengxinshui’ and ‘Cuiguan’ pears (Shi et al., 2021). Taken together, russeting formation may be a self-preservation mechanism in fruits. Thus, we analyzed the HSPs, AQPs, and PRXs involved in the response to biotic and abiotic stresses in the transcriptome data. We found three upregulated types of HSPs in russet apples (**Figure 6A**). Because HSPs respond to heat shock, higher HSP expression would increase heat tolerance in russet pears (Shi et al., 2021).

In addition, AQPs are non-enzymatic proteins that play roles in water stress by changing the cell wall structure and water channels of the cell membrane (Secchi et al., 2017). Three AQP genes, *PbTIP1-3*, *PbTIP2-1*, and *PbPIP2-5*, were highly expressed in rainfall-treated sand pears, indicating that these genes may be involved in rainfall-aggravated russeting (Shi et al., 2021). In our study, we also identified nine AQP-related genes that were differentially expressed in the two apple phenotypes. *PIP1* and *PIP2* were expressed at higher levels in russet peels and may play an important role in regulating water transport against moisture (**Figure 6A**). Russeting was accompanied by cuticle damage, which could easily lead to water loss. Therefore, the high expression of AQP genes in russet fruits could explain the cuticle damage caused by russet formation, which may accelerate fruit water loss. We found that most *PRX* genes were highly expressed in russet apples (**Figure 6A**). PRX has been suggested to play a role in auxin metabolism, lignification, suberization, cross-linking of cell wall components, and defense against pathogens (Quiroga et al., 2000). This result indicated that *PRX* genes promoted lignin and suberin synthesis, enhancing the response to stress in russet apples.

Plant hormones play an important role in fruit russeting, which can be reduced by hormone application (Knoche et al., 2011). However, their direct effect on fruit russeting is not clear; thus, the molecular mechanisms, by which hormones affect fruit russeting, should be studied in the future. A total of 216 DEGs were identified and involved in pear browning spot formation, including the IAA, CTK, GA, ABA, JA, and SA pathways, showing that GA might promote browning-spot formation (Wang et al., 2020). In pears, 19 DEGs related to plant hormone biosynthesis and signaling were identified, including ABA, GA, IAA, and ethylene. ABA content was higher in russet pears, and ABA treatment could increase russeting (Wang et al., 2022). Many genes related to auxin and ethylene signaling pathways are highly expressed in russet apples (Legay et al., 2015). Moreover, it is believed that there are fewer DEGs for salicylic acid and jasmonic acid in russet fruits, and there may not be a common wounding and biotic stress response (Legay et al., 2015). In this study, we found that the IAA, CK, ABA, BR, MeJA, and ethylene pathways were significantly different (**Figure 6B**). A large number of auxin pathway genes were enriched, including *IAA1*, *IAA7*, *IAA14*, *IAA19*, *IAA26*, *IAA27*, *IAA27*, *IAA31*, *ARF8*, and *AUX1*. In addition, we identified higher expression levels of the CK and ABA pathway genes *AHK3*, *AHK5*, and *PP2C2*. We found that genes of the IAA pathway were expressed in the highest numbers in russet fruits and speculated that IAA may play an important role in fruit russeting. In this study, we first discovered the existence of multilayered cell stacks in russet peels (**Figure 1D**). It is speculated that IAA and CK promote russeting by controlling cell differentiation and proliferation, while ABA and MeJA further promote secondary metabolism by participating in defense responses. Moreover, a large number of ethylene pathway genes were identified in this study, and the expression of various ERFs was downregulated; however, the role of this downregulation in fruit russeting is unclear. In addition, we found upregulated expression of *BSK* and *CYCD* genes in the BR pathway. The relationship between BR and fruit russeting remains uncertain, which provides a new idea for future research.

### Transcription factors involved in fruit russeting

Although fruit russeting involves metabolic synthesis and is influenced by the environment, transcription factor regulation is also important in russet formation. In plants, various MYB transcription factors regulate either suberin biosynthesis or extracellular lipid accumulation. In pears, 12 MYB genes involved in plant tissue suberization were differentially upregulated in the russet fruit skin, indicating that these MYB genes may affect russeting formation by regulating suberin synthesis (Ma et al., 2021). In addition, MYB9 and MYB107 (Lashbrooke et al., 2016), MYB6 and MYB67 (Falginella et al., 2021), and MYB93 (Legay et al., 2016) involvement has been reported in suberin in fruit russeting. Interestingly, several upregulated MYB transcription factors were identified in this study. In addition to transcription factors whose synthesis has previously been reported in fruit russeting and suberin, we found that MYB73 and MYB84 also had high levels of differential expression.

WRKY transcription factors can bind to stress-related promoters to regulate plant tolerance to abiotic stresses. According to the transcriptome results, we identified 31 WRKY transcription factors, only seven of which were downregulated. Among them, WRKY13 expression was the highest and its expression was higher in russet apple than that in non-russet apple (**Figure 5E**). AtWRKY13 was identified as a positive factor for lignin synthesis, and *AtWRKY13* overexpression could improve the mechanical strength of *Arabidopsis* stems (Li et al., 2015). Thus, WRKY13 may be a positive factor for lignin synthesis during russeting.

## Conclusion

In this study, the high lignin and suberin content led to russet formation in the skin of the russet apple strain. Transcriptome and metabolome analysis revealed that the expression of suberin and lignin synthesis genes was upregulated in the skin of russet apples, and the intermediate metabolites involved in both pathways were abundantly accumulated. These results indicate a positive correlation between lignin and suberin synthesis and fruit russeting. Taken together, lignin and suberin accumulation in fruit skin is an important reason for fruit russet formation.

## Data availability statement

The original contributions presented in the study are publicly available. This data can be found here: NCBI, PRJNA871277.

## Author contributions

SJ and YZ designed this experiment and revised the manuscript, ZW and SJ analyzed the data and wrote the manuscript. SL and WH helped to perform the qRT-PCR; MC helped ZW to analyzed the transcriptome data. All authors contributed to the article and approved the submitted version.

## Funding

This work is supported by the National Natural Science Foundation of China (32102319), Natural Science Foundation of Shandong Province (ZR2021QC010), National Key Research and Development Program Foundation (2019YFD1001403), Taishan Scholar Foundation of Shandong Province (ts2022), Agricultural Variety Improvement Project of Shandong Province (2021LZGC024), Talents of High-level Scientific Research Foundation (6651121002) and Graduate Innovation Program of Qingdao Agricultural University (QNYCX21089).

## Conflict of interest

The authors declare that the research was conducted in the absence of any commercial or financial relationships that could be construed as a potential conflict of interest.

## Publisher’s note

All claims expressed in this article are solely those of the authors and do not necessarily represent those of their affiliated organizations, or those of the publisher, the editors and the reviewers. Any product that may be evaluated in this article, or claim that may be made by its manufacturer, is not guaranteed or endorsed by the publisher.

## Abbreviations

DEGs, differentially expressed genes; DAFB, days after full bloom; SEM, scanning electron microscopy; GO, Gene Ontology; KEGG, Kyoto Encyclopedia of Genes and Genomes; DAMs, differentially accumulated metabolites; WGCNA, weighted gene co-expression network analysis; CK, cytokinin; ABA, abscisic acid; BR, brassinolide; MeJA, methyl jasmonate; HSPs, heat shock proteins; AQPs, aquaporins; PRXs, peroxidases.
