# Supplementary material for: Transcriptome and metabolome analyses reveal phenotype formation differences between russet and non-russet apples
Source: Front Plant Sci. 2022 Nov 8;13:1057226. doi: 10.3389/fpls.2022.1057226 (PMC9678910; doi:10.3389/fpls.2022.1057226)
Supplement: Supplementary file 2 [file DataSheet_2.docx]

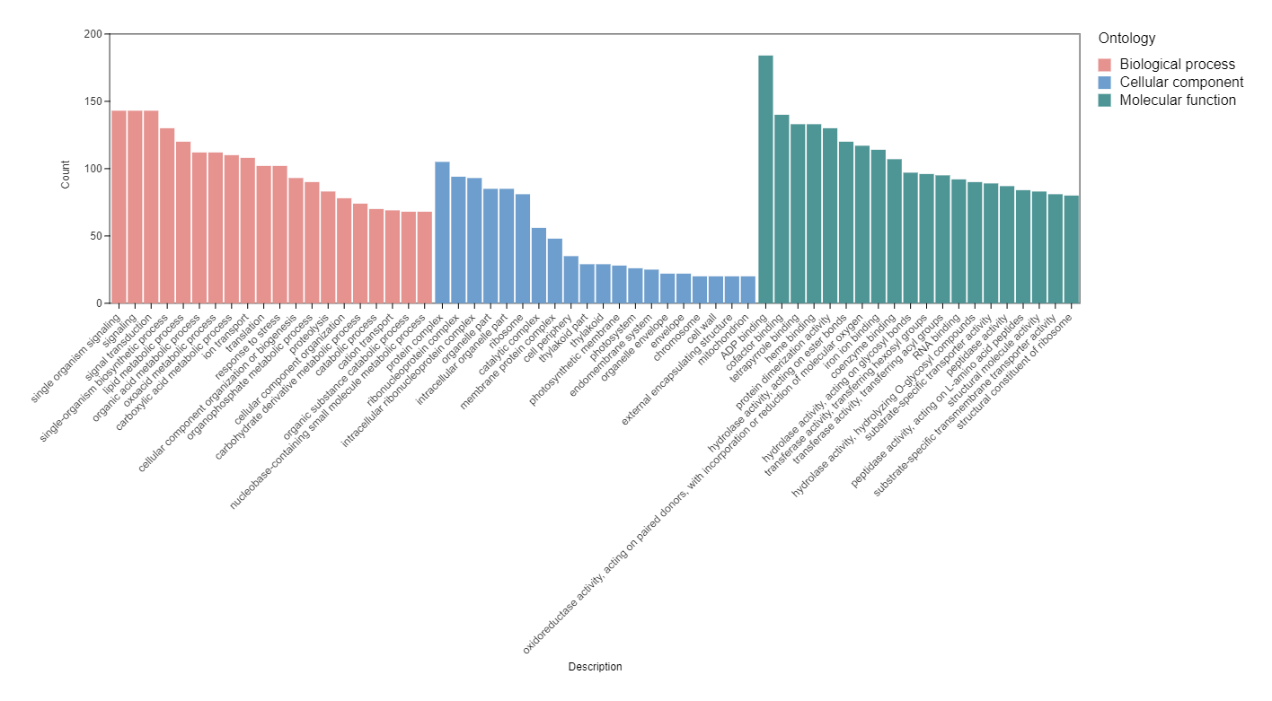


Figure S1. GO classification of the DEGs identified under the biological process, molecular function, and cellular component categories.


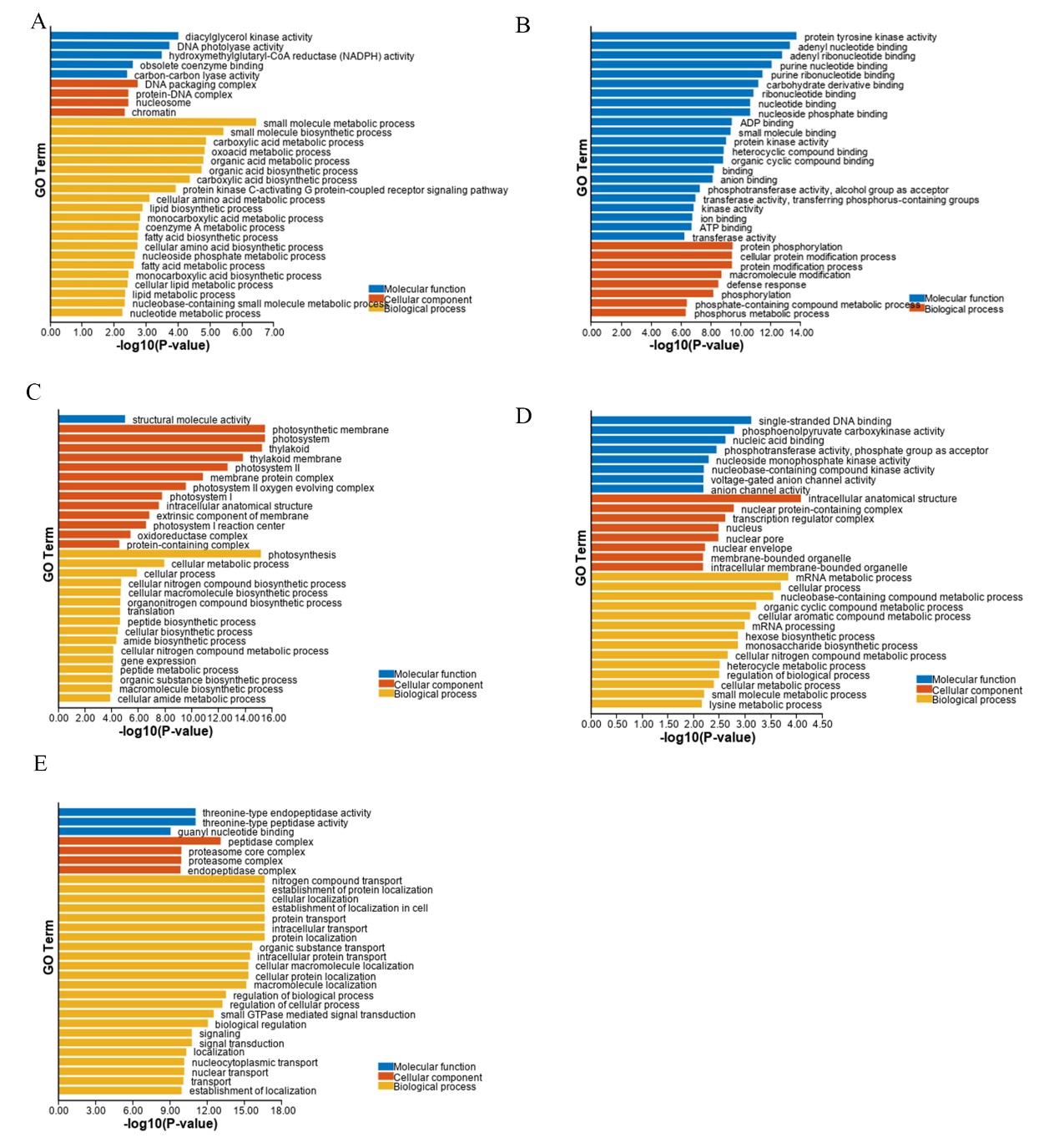


Figure S2. GO enriched in blue (A), blue2 (B), mediumorchid (C), black (D) and blue4 (E) modules.
